# Hearing Screening-Driven Investigation of Newborns for Congenital Cytomegalovirus Infection at a German University Hospital

**DOI:** 10.3390/ijns12030052

**Published:** 2026-07-08

**Authors:** Niko Kohmer, Lena Mistry, Thorsten Mosler, Sabine Kramer, Annette Weiß, Alfred Lennart Bissinger, Nora Doberschuetz, Ulrich Rochwalsky, Holger F. Rabenau, Horst Buxmann

**Affiliations:** 1 Institute of Medical Virology, University Hospital Frankfurt, Goethe-University Frankfurt am Main, 60590 Frankfurt am Main, Germany; niko.kohmer@ukffm.de (N.K.); rabenau@em.uni-frankfurt.de (H.F.R.); 2 Princess Margaret Children’s Hospital Darmstadt, 64287 Darmstadt, Germany; lenakmistry@gmail.com; 3 Institute of Biochemistry II, Goethe-University Frankfurt am Main, 60590 Frankfurt am Main, Germany; 4 Section of Phoniatrics and Paediatric Audiology, Evangelisches Krankenhaus Oldenburg, Universitätsmedizin, 26122 Oldenburg, Germany; sabine.kramer@uni-oldenburg.de; 5 Section of Phoniatrics and Paediatric Audiology, ENT Clinic, University Hospital Frankfurt, Goethe-University Frankfurt am Main, 60590 Frankfurt am Main, Germany; 6 Institute of Medical Virology and Epidemiology of Viral Diseases, University Hospital Tübingen, 72076 Tübingen, Germany; alfred.bissinger@med.uni-tuebingen.de; 7 Department of Pediatrics, Division of Neonatology, Goethe-University Frankfurt am Main, 60590 Frankfurt, Germany; nora.doberschuetz@unimedizin-ffm.de (N.D.); ulrich.rochwalsky@unimedizin-ffm.de (U.R.); 8 Department of Paediatrics and Neonatology, Main-Kinzig Clinics Gelnhausen, 63571 Gelnhausen, Germany

**Keywords:** congenital CMV, newborn hearing screening, hearing impairment, cCMV screening

## Abstract

Congenital Cytomegalovirus (cCMV) infection is the leading non-genetic cause of sensorineural hearing loss in newborns. Systematic nationwide screening programmes are lacking. Antiviral valganciclovir therapy could improve auditory outcomes if initiated within the first 30 days of life, making timely diagnosis crucial. To address this, we investigated whether a hearing screening-based protocol is suitable. Between 2015 and 2019, newborns, aged ≤21 days, with repeated abnormal newborn hearing screening (NHS) were prospectively enrolled at University Hospital Frankfurt. Oral mucosal swabs were tested for CMV DNA by real-time PCR, with confirmatory urine and blood diagnostics in positive cases. Of 2741 infants presenting for repeat NHS, 2059 (75.1%) showed normal bilateral findings. Of the 682 (24.9%) with abnormal results, 575 (84.3%) were aged >21 days and thus ineligible. A total of 107 infants (3.9%) met both criteria—abnormal NHS and aged ≤21 days—of whom 100 entered per-protocol analysis. Two (2%) were confirmed as cCMV-positive and received valganciclovir. Among the 48 infants who additionally underwent DBS testing, diagnostic sensitivity and specificity were 100%. The presented NHS-driven cCMV protocol reliably identified cCMV-infected newborns to offer timely antiviral therapy. In the absence of universal cCMV screening, this targeted approach offers a challenging but WHO screening-criteria-compliant strategy to enable timely antiviral intervention.

## 1. Introduction

Human cytomegalovirus (CMV), also known as Human Herpesvirus 5, is an enveloped double-stranded DNA virus that was independently discovered in 1956 and 1957 [1,2]. It is the most common congenital infection in developed countries, affecting 0.2–0.6% of live births in Germany and approximately 7 out of 1000 births worldwide [3,4]. CMV persists for life in lymphocytes and other cell populations, with seroprevalence rates of up to 60% in industrialised nations and nearly 100% in developing countries, and is mostly dependent on socioeconomic status [5,6].

Congenital CMV (cCMV) infection results from vertical transmission during pregnancy, with 14–52% of newborns from mothers with primary infection during pregnancy becoming intrauterine infected, depending on the time of primary infection, age, and socioeconomic status of the women [7,8]. In Germany, the CMV transmission rate is 40% in relation to the whole pregnancy [9]. Reinfections with different strains or reactivations (10–20% of CMV-positive mothers) show much lower transmission rates of up to 2% [10,11]. Although 90% of congenitally infected children are initially clinically asymptomatic, CMV causes significant morbidity and mortality [6]. Severe manifestations include microcephaly, intracranial calcifications, hepatosplenomegaly, and intrauterine growth restriction. Most notably, sensorineural hearing loss (SNHL) is the most common sequela, occurring in 5–20% of asymptomatically infected infants and 30–65% of symptomatic cases at birth [12,13,14,15]. Given the frequently progressive nature of SNHL attributable to cCMV, interventions directed towards hearing preservation are of paramount importance.

cCMV diagnosis relies primarily on polymerase chain reaction (PCR) amplification of CMV DNA from urine within the first 21 days of life. Whilst oral secretions are more readily obtained and facilitate the identification of CMV-infected neonates, sampling must strictly be performed within this 21-day-of-life window in order to distinguish congenital from postnatal infection (e.g., transmitted via breast milk) [16,17]. Beyond three weeks of age, when viral shedding in urine or saliva can no longer reliably confirm the timing of infection, the diagnosis of cCMV must be established retrospectively—by means of CMV PCR analysis of dried blood spot (DBS) cards, for example. Serological testing for CMV-specific IgG and IgM antibodies and viral cultures on fibroblasts are alternative diagnostic methods. Ganciclovir and its oral prodrug valganciclovir are the mainstay antivirals for symptomatic congenital CMV disease, with evidence of improved long-term hearing preservation and neurodevelopmental outcomes, and could be started as off-label therapy within the first month of life [18,19,20].

Despite the substantial clinical burden of congenital CMV-associated hearing loss, no CMV active vaccine is currently available for prevention. Notably, a few US states have implemented hearing screening-driven cCMV programmes since 2013 (Utah) and 2016 (Connecticut), identifying affected infants who may benefit from early antiviral intervention [21]. Since 1 July 2025, DBS testing for cCMV has become mandatory for all newborns born in Connecticut [22]. Germany currently lacks such a systematic screening programme.

This study aims to detect cCMV infection in infants with repeated newborn hearing screening (NHS) failures and to evaluate whether protocol-based screening—using transiently evoked otoacoustic emissions (TEOAEs) and automated auditory brainstem response (AABR), followed by laboratory cCMV confirmation—can identify symptomatically infected newborns eligible for antiviral therapy in a timely manner.

## 2. Patients and Methods

### 2.1. Patients

Eligible participants included newborns aged ≤3 weeks who attended the Department of Paediatric Audiology at University Hospital Frankfurt between 3 August 2015 and 31 December 2019 for rescreening following an initially failed hearing screening via TEOAEs or AABR, and who demonstrated a further abnormal result on repeat hearing screening. Written informed consent was obtained from parents or legal guardians. Exclusion criteria were ages >3 weeks, normal findings at hearing screening, and a known or previously excluded cCMV infection.

For enrolled infants, a buccal swab for CMV DNA testing was collected by a medical professional, rotating a sterile cotton swab in each buccal pouch for approximately 3 s. To minimise contamination with CMV-containing breast milk, sampling was performed at least 30 min after the last milk feeding [23]. Swabs were analysed by CMV PCR at the Institute for Medical Virology, University Hospital Frankfurt.

Newborns with a positive CMV PCR result underwent confirmatory diagnostics, including clinical examination (length, weight, head circumference), cranial ultrasound, CMV PCR and virus isolation and culture from urine, and blood tests (complete blood count, clinical chemistry [AST, ALT, GLDH, total and direct bilirubin, creatinine, urea], CMV serology, and CMV PCR).

With parental consent, the DBS card was requested from the regional screening centre. If still available (i.e., not destroyed according to national regulations), the dried blood spots (DBSs) were tested for CMV DNA by PCR at the German consultation laboratory for congenital CMV infection in the virology department of University Hospital Tuebingen, Germany.

**Figure 1:**
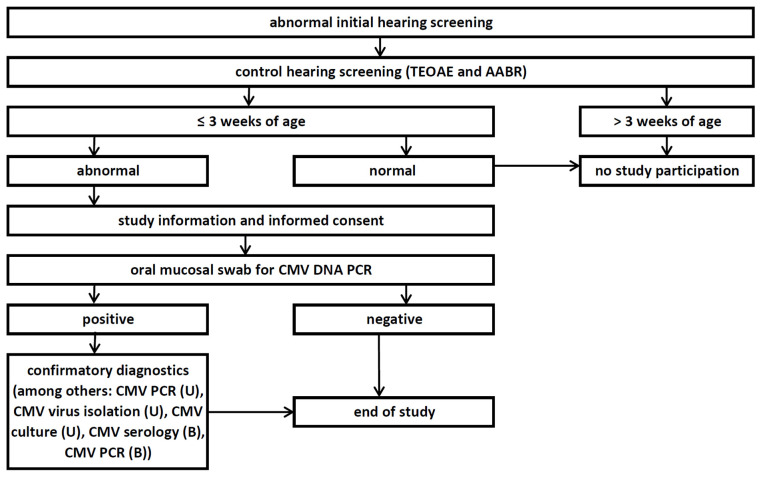
Study flowchart (U = urine; B = blood).

The following data were collected for all enrolled infants: name, date of birth, dates and results of hearing screening and buccal swab, any known CMV status, CMV PCR result from the buccal swab, results of confirmatory diagnostics, and CMV PCR result from the DBS card (Figure 1).

### 2.2. German Universal Newborn Hearing Screening (NHS) Programme

During the study period, the German universal newborn hearing screening (NHS) programme was governed by the resolution of the Federal Joint Committee (Gemeinsamer Bundesausschuss, G-BA) of 19 June 2008 [24], which came into force on 1 January 2009 and remains valid to date. The screening protocol is stipulates as follows.

- Screening comprises measurement of transient evoked otoacoustic emissions (TEOAEs) and/or automated auditory brainstem response (AABR), with screening success contingent upon result reliability and timely comprehensive paediatric audiological follow-up in cases of abnormal findings.
- Each ear is tested by TEOAEs or AABR no later than day 3 of life. AABR is mandatory for infants at risk of congenital hearing loss. Hospital-born infants are screened prior to discharge; infants born outside hospital are screened no later than at the second routine paediatric examination (‘U2’—which is done between days of life 3 and 10).
- A failed initial TEOAE or AABR result requires a control AABR of both ears, ideally on the same day, and no later than the ‘U2’ examination.
- A failed control AABR necessitates comprehensive paediatric audiological confirmatory diagnostics by the 12th week of life.

### 2.3. Transiently Evoked Otoacoustic Emissions (TEOAEs)

TEOAEs are low-level sounds emitted by outer hair cells in the cochlea in response to acoustic stimuli. These emissions are captured by a sensitive microphone in the ear canal. They typically cover the main speech frequency range from 0.5 to 4 kHz. The presence of TEOAEs indicates that the outer hair cells of the cochlea are functioning correctly and that the middle ear is clear, which is essential for normal sound amplification. Since these emissions generally disappear when hearing loss exceeds 30 to 35 dB HL, their positive detection suggests that hearing thresholds are within a normal or near-normal range.

TEOAEs were recorded with the Echo-Screen TDA system (Fischer-Zoth Diagnosesysteme GmbH; distribution: Mack Medizintechnik GmbH, Pfaffenhofen an der Ilm, Germany) at the Department of Paediatric Audiology, University Hospital Frankfurt. Each ear was tested sequentially using an ear-canal probe, integrating a click transducer and microphone, coupled to the external auditory canal via a silicone adapter to ensure an airtight seal. The device delivered a non-linear click sequence at 70–85 dB SPL (self-calibrated, depending on ear-canal volume) at an approximate repetition rate of 60 Hz. Cochlear responses were evaluated in the 6–12 ms post-stimulus interval. Response detection and pass/refer classification were performed automatically by the device, using its built-in binomial statistical algorithm to test for the presence of a stimulus-related response, based on eight test points and parallel analysis across eight quality groups. A response was considered present when the probability for a random distribution fell below 0.3% (significance 99.7%); otherwise, the device returned a “refer” result if the criterion was not met after 200 scored stimuli. Measurements were conducted in a low-noise setting.

### 2.4. Automated Auditory Brainstem Response (AABR)

AABR screening is based on the auditory brainstem response (ABR), as described by Jewett and Williston in 1971 [25]. The ABR comprises a series of far-field electrophysiological waveforms generated along the auditory pathway within the first approximately 10 ms following acoustic stimulation. Typically, five to seven waves (I–VII) can be identified, with wave V being the most robust component in neonates and therefore primarily used for automated detection. The detection of wave V in the AABR proves a proper transmission of auditory information to the brainstem and indicates regular retrocochlear function.

Bilateral AABR screening was performed using the Echo-Screen TDA device (Fischer-Zoth Diagnosesysteme GmbH; distribution: Mack Medizintechnik GmbH, Pfaffenhofen an der Ilm, Germany). Each ear was tested sequentially using an ear-canal probe. Disposable gel electrodes were positioned with the non-inverting (active) electrode on the high forehead and electrodes on both mastoid processes. During monaural testing, responses were recorded differentially between the forehead and the ipsilateral mastoid (ears tested sequentially). The electrode on the mastoid of the contralateral (non-tested) ear was used as the reference. Electrode impedance was verified by the device and maintained below 12 kΩ. Acoustic stimulation consisted of a 35 dB nHL broadband click presented at an approximate repetition rate of 55 Hz. Responses were sampled at 10.2 kHz and evaluated using an analysis interval of 170 samples (17 ms) with the device’s built-in binomial statistical detection algorithm; a “pass” was assigned when the predefined 99.5% significance criterion was reached.

### 2.5. CMV PCR

Oral mucosa swabs were analysed at the Institute for Medical Virology, University Hospital Frankfurt, using an in-house real-time PCR assay (accredited according to DIN EN ISO 15189, https://www.dinmedia.de/de/norm/din-en-iso-15189/375920985, accessed on 17 April 2026). Nucleic acids were extracted with the QIAsymphony system and DSP virus/pathogen midi kit (Qiagen GmbH, Hilden, Germany). CMV DNA concentration was quantified on the ABI PRISM^®^ 7500 analyser (Applied Biosystems, Waltham, MA, USA) with TaqMan Gene Expression Master Mix (Thermo Fisher Scientific, Darmstadt, Germany), targeting the UL89 gene. Oral mucosa swabs were evaluated semi-quantitatively using Ct values, whereas CMV DNA concentrations were quantified in urine, serum, and plasma samples. As an internal control, murine CMV virions (strain Smith; ATCC VR-1399) were used [26]. Viral concentrations were calibrated to the CMV World Health Organization (WHO) International Standard and reported as IU/mL (limit of detection: 200 IU/mL). For confirmation diagnostics, CMV DNA PCR testing was additionally performed on urine (minimum 0.8 mL) and EDTA plasma (minimum 1.6 mL). Prior to testing, swabs and urine were diluted with an equal volume of phosphate-buffered saline (PBS). This dilution step was omitted for plasma testing.

The DBSs for CMV DNA were sent to the German consultation laboratory for congenital CMV infection in the virology department of University Hospital Tuebingen, Germany. There, an entire dried blood spot (13 mm) was analysed using quantitative real-time PCR (values in copies/mL) and additionally nested PCR (qualitative) according to previously published methods [27].

### 2.6. Anti-CMV IgM and IgG Testing

Serum samples were analysed for anti-CMV IgM and IgG using the Enzygnost^®^ anti-CMV IgM and IgG assays (Dade Behring, Marburg, Germany) on the Behring ELISA Processor BEP 2000 (Siemens Healthineers AG, Erlangen, Germany), in accordance with the manufacturer’s instructions. The results were calculated using the alpha method by subtracting the control antigen value in the Behring ELISA Processor. CMV IgM and IgG results are reported semi-quantitatively as arbitrary units per millilitre (AU/mL).

### 2.7. CMV Isolation

The shell vial assay was used for rapid CMV detection from fresh urine (<16 h) on foreskin fibroblasts according to an already established in-house protocol [28,29]. Infected cells were microscopically counted and averaged. Results were valid only if negative controls showed no staining and positive controls showed specific red–brown nuclei.

## 3. Results

### 3.1. Study Population and Screening Characteristics

**Figure 2:**
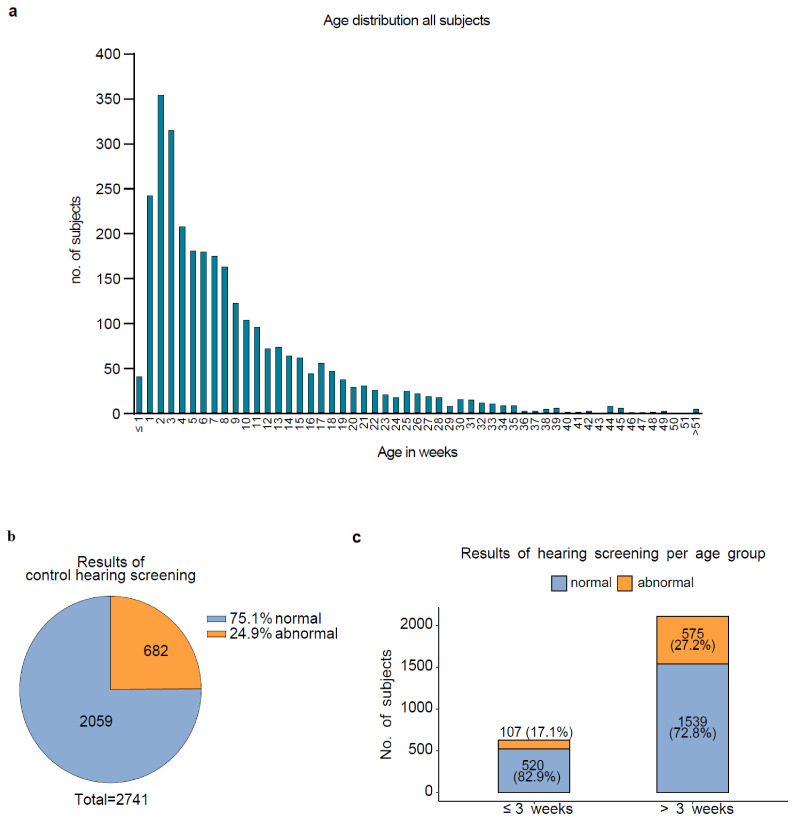
Study population and hearing screening results: (**a**) age distribution of patients at the time of the control hearing screening; (**b**) results of control hearing screening; (**c**) results of hearing screening per age group.

**Figure 3:**
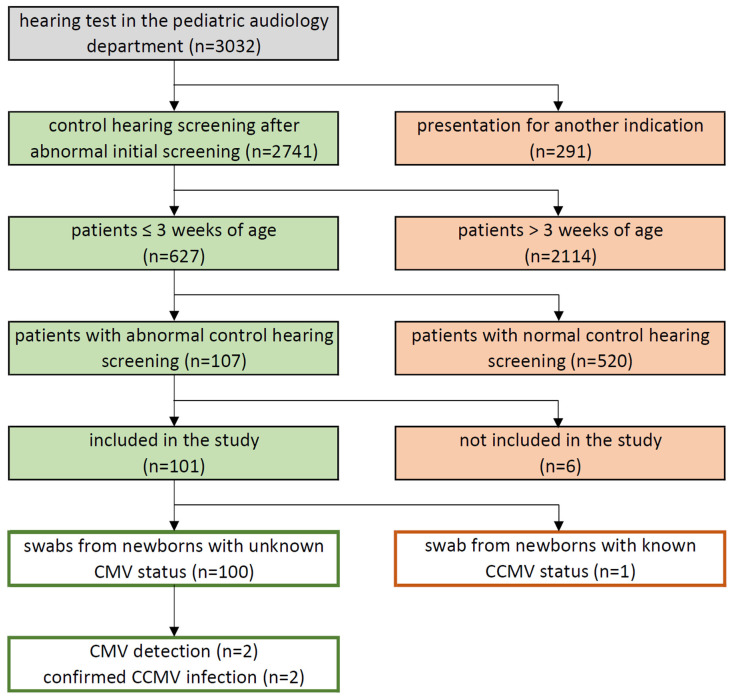
Overview of patient presentations in paediatric audiology. Six infants received CMV swabs despite meeting exclusion criteria and were therefore excluded from per-protocol analysis: two were older than 21 days, one had an unaffected control screening, one had a previously excluded cCMV infection status, one presented for control screening after an unaffected initial screening with known cCMV, and one underwent initial screening at home birth not captured in the 2741-patient cohort.

From 3 August 2015 to 31 December 2019, the Department of Paediatric Audiology at University Hospital Frankfurt recorded 3032 appointments as the control for hearing screening. Of these, 291 (9.6%) were for non-study indications (e.g., post-ototoxic therapy), while 2741 (90.4%) patients presented for initial repeat screening after a failed first NHS test, constituting our study cohort (Figure 2 and Figure 3).

Mean age at repeat screening was 70 days (median 46); 2114 children (77.1%) were >3 weeks old. Thus, 627 infants (22.9%) remained within the 3-week diagnostic window (Figure 3) for cCMV if repeat screening failed. Age distribution is shown in Figure 2a.

In total, 2059 of all 2741 analysed infants (75.1%) showed normal results bilaterally in the control hearing screening, both in the TEOAEs and AABR tests. Meanwhile, 682 patients (24.9%) had at least one abnormal finding in the control hearing screening (Figure 2b).

Of the 682 patients with abnormal control hearing screening, 575 (84.3%) were older than 21 days and thus ineligible for study participation. Only 107 of the 2741 infants (3.9%) showed abnormal confirmatory screening while remaining within the eligible age window of ≤21 days (Figure 2c and Figure 3).

### 3.2. Study Enrollment and CMV Sample Collection

Six of these 107 eligible infants were not enrolled in the study. Two had a previously documented cCMV status (exclusion criterion met). Two infants presented with unilateral ear canal stenosis, which was incorrectly classified as an exclusion criterion. Two additional candidates could not be adequately enrolled due to language barriers in parental counselling.

### 3.3. Patient Characteristics and Hearing Screening Results

**Figure 4:**
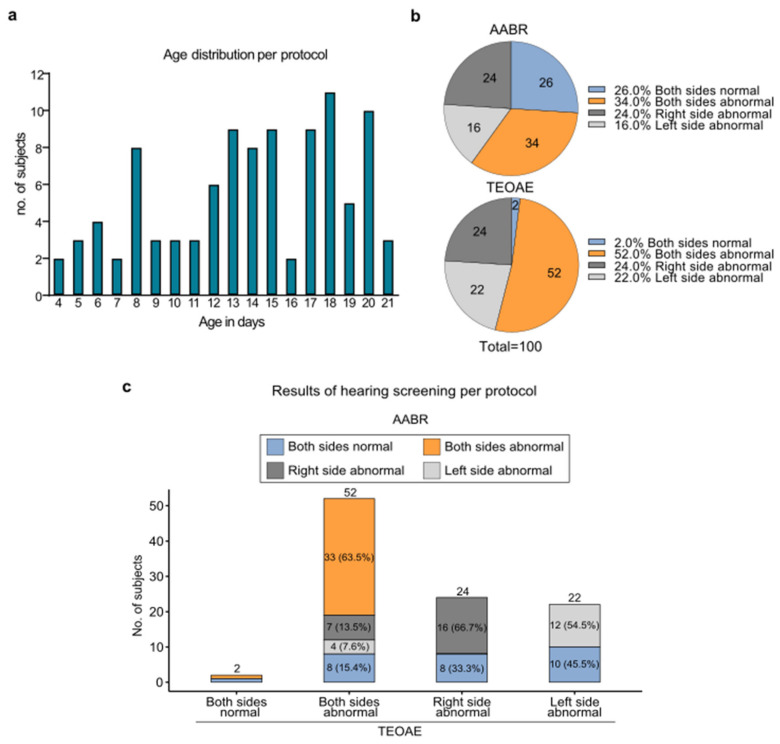
Patient characteristics and hearing screening results: (**a**) age distribution of patients; (**b**) hearing screening results according to AABR and TEOAEs; (**c**) overall hearing screening results.

The per-protocol cohort comprised 100 infants who met all inclusion criteria. Participants had a mean age of 14 days at enrolment, with the youngest participant 4 days old and the oldest 21 days old. Age distribution is shown in Figure 4a.

Hearing screening results are presented in Figure 4b,c. Most infants showed bilateral abnormalities in both TEOAE (52%) and AABR (34%) testing (overall: 63.5%), whereas a minority exhibited abnormalities solely in AABR.

### 3.4. CMV Oral Mucosal Swabs Results

Of the 100 oral mucosal swab samples analysed, 98 (98%) yielded negative CMV PCR results, while two (2%) were CMV PCR-positive. The Ct values for CMV-positive samples averaged 21.2.

### 3.5. Confirmatory Diagnostics

Both CMV PCR-positive patients in oral mucosal swabs underwent confirmatory diagnostics with concordant positive results (100% concordance). Detailed laboratory parameters, including CMV DNA in oral mucosal swabs (Ct values), CMV IgG and CMV IgM serology, CMV isolation from urine, CMV DNA in plasma, CMV DNA in urine, and CMV DNA in DBSs, as well as the results of the hearing tests, are presented in Table 1.

### 3.6. DBS Card Results

DBS cards were obtained and tested for CMV DNA in 48 of the 100 per-protocol participants (48%). Of these, two cards (4.2%) tested CMV-positive and 46 (95.8%) tested CMV DNA-negative. Concordance between DBS card results and oral mucosal swab results was achieved in 46 cases (95.8%). One positive CMV PCR result from a DBS card was classified as a false-positive, as both confirmatory diagnostics and the corresponding oral mucosal swab provided no evidence of cCMV infection. One DBS card initially tested negative in automated real-time PCR; however, upon testing using the nested PCR, CMV DNA was successfully detected. One DBS card test had to be excluded from the per-protocol analysis because the corresponding newborn presented for the initial screening rather than for a repeat hearing screening in the Department of Paediatric audiology.

### 3.7. Diagnostic Accuracy: Sensitivity and Specificity

Among the 48 per-protocol participants who underwent multiple independent CMV tests, all metrics yielded 100% sensitivity (2/2), specificity (46/46), positive predictive value (2/2), and negative predictive value (46/46).

### 3.8. Clinical Outcome

In summary, our study protocol successfully identified symptomatic cCMV infection in two infants within the critical diagnostic window of 21 days or fewer. This was achieved through targeted screening prompted by abnormal newborn hearing screening results in a preselected cohort of infants with undetermined hearing status and unknown cCMV infection status. Both patients received off-label antiviral therapy with valganciclovir. In patient 1, hearing impairment in a low-frequency notch on the right ear remained stable, below the threshold for hearing aid fitting until the age of one year. At that time, according to the Bayley Scales of Infant and Toddler Development, Third Edition (Bayley-III), he demonstrated cognitive development and fine and gross motor skills within the average range, whilst language performance was rated slightly above the age-equivalent norm. In patient 2, high-frequency hearing loss with a notch of up to 45 dB in the left ear normalised within the first two years of life. At that age, assessment using the Bayley Scales of Infant and Toddler Development, Third Edition (Bayley-III), demonstrated cognitive development and gross motor skills within the age-appropriate range, whilst fine motor skills and language performance were rated slightly above the age-equivalent norm. No further clinical findings could be observed in these children.

## 4. Discussion

This study demonstrates that a protocol-based, hearing screening-driven approach to cCMV diagnosis is highly effective in identifying newborns with cCMV-associated hearing loss within the critical diagnostic window of the first 21 days of life. The presented protocol thereby affords the opportunity to complete the recommended cCMV diagnostic workup in affected neonates and to offer antiviral therapy against cCMV within the recommended first 30 days of life.

However, as the majority of infants in our cohort had to be excluded owing to advanced age at paedaudiological presentation, systematic improvements to the newborn hearing screening pathway are urgently required to enable timely cCMV diagnosis. Consequently, the current German guideline on the prevention and management of cCMV infection (in effect since November 2024) stipulates that newborns with a repeated abnormal universal newborn hearing screening (NHS) result should undergo prompt urinary cCMV-DNA testing at their birth centre [30].

Among 100 protocol-eligible infants with repeated abnormal NHS, 2% were confirmed to have cCMV infections. The cCMV detection rate of 2% in the present targeted cCMV screening study exceeds the prevalence of congenital hypothyroidism (~0.035%), the most common disorder included in standard universal neonatal metabolic screening, which is approximately 60-fold. This highlights the substantial clinical burden of cCMV-associated hearing loss in the investigated targeted population. Due to the strict inclusion criteria of our study, the presented results do not reflect the incidence of cCMV in newborns with hearing impairment, which is even higher [31].

The diagnosis of cCMV infection was made in the two identified children by CMV DNA detection using their buccal swabs, with Ct values of 25 and 17, confirmed by CMV DNA concentrations of 61 and 34 × 10^6^ IE/mL in their urine. The CMV DNA concentrations in blood were quite low at 200 and 2.270 IU/mL. Interestingly, the CMV DNA results in the DBS samples were inversely proportional, with 2.430 copies/mL and non-quantifiable, as shown in Table 1. The serological findings in both children were not suggestive for cCMV infection: both newborns had positive CMV IgG and negative CMV IgM levels. These findings underscore the high importance of diagnosing cCMV infection by using urine and mucosal swab samples [30,32].

The hearing impairments detected in the two children with cCMV infection during NHS were confirmed in subsequent paediatric audiological follow-up examinations as unilateral hearing loss in each case. Both patients received antiviral therapy with valganciclovir. The hearing impairment in patient one remained stable, below the threshold for hearing aid fitting, while in patient two, hearing function normalised completely over time during antiviral therapy. As part of the follow-up examinations, patient one was last presented the Bayley-III test at the age of one year. Here, he demonstrated normal cognitive, gross, and fine motor development, as well as a developmental lead of 4 months in the linguistic domain. Patient two was last seen for follow-up examination at the age of 6 years and presented with somatic, cognitive, and neurological development entirely appropriate for their age, including normal hearing function.

A central strength of the presented protocol is its alignment with the WHO screening criteria, as defined by Wilson and Jungner [33]. cCMV infection represents a well-defined and clinically important condition. It can be reliably detected through validated molecular diagnostics (CMV PCR from oral mucosal swabs and urine), and an accepted, effective treatment exists in the form of parenteral ganciclovir or oral valganciclovir, which has been shown to improve long-term hearing and neurodevelopmental outcomes when initiated within the first 30 days of life [18,19,34]. The natural history of the disease is well-understood, and the primary diagnostic test itself is simple, non-invasive, and accurate. In our small per-protocol cohort, for example, the CMV diagnostic achieved 100% sensitivity and specificity, which is in accordance with the extensive studies on cCMV diagnosis by mucosal swabs published by Boppana et al. 2010 and 2011 [35,36]. It must be acknowledged, however, that cCMV-negative results in our small cohort were not subjected to further confirmatory testing. Meanwhile, it had been shown that mucosal swabs taken to diagnose cCMV infection can result in false-positive findings due to CMV shed in breast milk [37]. Therefore, it is crucial to adhere to both the post-feed sampling interval and the required cCMV confirmation diagnostics of our protocol, especially for CMV DNA analysis in urine.

Taken together, the presented full investigation plan fulfils the essential requirements for a justified screening intervention, as the illness is important, can already be detected at a clinically pre-symptomatic or early symptomatic stage, and is treatable with meaningful clinical benefit.

cCMV infection represents a causally treatable aetiology of congenital sensorineural hearing loss, whereas in the most other causes, only rehabilitative approaches, such as hearing aids or cochlear implants, could be offered. Antiviral off-label therapy with enteral valganciclovir offers an opportunity to address the underlying infectious process and preserve or improve auditory function. In particular, this applies to mildly symptomatic cCMV-infected neonates identified through our screening protocol, as the likelihood of hearing stabilisation or even improvement correlates inversely with the severity of hearing loss [38]. Conversely, profound congenital hearing loss rarely demonstrates improvement with antiviral therapy [18,20]. For affected families, early identification and timely treatment initiation are therefore of high value—both medically and personally. Preventing hearing loss is highly cost-effective. It minimises expenditures for auditory rehabilitation (e.g., expensive medical devices like CIs, hearing aids), specialised support services, and specialised schooling, while facilitating long-term socioeconomic integration. Unaddressed hearing loss causes annual costs of nearly one trillion US dollars. The WHO postulates a return on investment (ROI) of 1:16 for investments in hearing care [39,40]. Due to its critical importance, the universal newborn hearing screening (UNHS) was established in Germany by an amendment to the Paediatric Guidelines (Kinder-Richtlinien) by the Federal Joint Committee (G-BA) in June 2008 [24]. Because approximately 21% of all permanent bilateral hearing loss in children at the age of four is attributable to cCMV infection specifically [41], investment in the prevention, early detection, and treatment of cCMV mitigates long-term socioeconomic burdens by reducing reliance on specialised education and medical devices while fostering economic productivity through lifelong contributions to social security. Beyond hearing loss, cCMV causes neurodevelopmental sequelae, such as cerebral palsy, microcephaly, and visual impairment, necessitating lifelong care and costly interventions. These comorbidities further escalate the socioeconomic burden by requiring extensive specialised support and long-term assistance. Several studies state that the costs for screening and early intervention are marginal compared to the gains in cognitive capacity and economic productivity over the entire lifespan [21,42,43,44]. This makes the secondary screening protocol presented here scientifically and clinically very precious, although a universal screening would be more cost-effective in prevention and early treatment [45]. Shahar-Nissan et al. (2020) showed that early treatment after CMV primary infection in the first trimester of pregnancy can reduce the risk of vertical CMV transmission by approximately 70% [46].

In summary, universal cCMV screening at birth would identify a substantially higher proportion of infected neonates than targeted screening, as the majority of cCMV-infected newborns are asymptomatic at birth and would not meet the referral criteria of a hearing-targeted protocol [17,47,48]. However, universal screening would pose considerable logistical and economic challenges, especially because urine is the best body fluid to diagnose cCMV infection. Current evidence does not seem to support its routine implementation yet [16,49]. Targeted screening, linking cCMV testing to a failed universal newborn hearing screening (UNHS) result, would represent a pragmatic and cost-effective alternative, albeit one that would inherently miss asymptomatic cCMV-infected infants with normal hearing at birth who may develop late-onset hearing loss [50]. Arguably, however, the most cost-effective, best-tolerated, and ultimately most impactful intervention would be the primary prevention of cCMV infection itself through the implementation of hygiene-based behavioural measures—particularly hand hygiene and avoidance of contact with young children’s saliva and urine—among CMV-seronegative women of reproductive age with active family planning, necessitating routine CMV serological screening in this population [51,52,53]. Regrettably, the majority of women of childbearing age remain insufficiently informed about cCMV infection [54,55]—the most common congenital infection—including the simple yet effective hygiene measures that can substantially reduce the risk of primary infection [51,52,53]. This critical shortcoming highlights the important role of gynaecologists in raising awareness of cCMV infection in women of childbearing age. An inevitable limitation of our study is the strict inclusion criterion of an age ≤ 3 weeks at repeated failed NHS. Since infants at age > 3 weeks were not tested, hearing loss caused by cCMV may not have been captured. Consequently, this leads to an underestimation of the proportion of cCMV-related hearing loss within the overall population. In our study, 575 newborns with repeated failed NHS (84.3%) were older than 21 days and thus ineligible for study participation. Only 107 of the 2741 infants (3.9%) showed abnormal confirmatory screening while remaining within the eligible age window of ≤21 days.

Therefore, the presented data also highlight a significant systemic challenge: 77.1% of infants presenting for repeat NHS were older than 21 days at the time of their appointment—the upper age limit for reliable cCMV diagnosis from fresh samples. Thus, the vast majority of children with potential cCMV-related hearing loss are referred too late for the diagnostic and therapeutic window to be exploited. This finding underscores an urgent need for structural improvements in NHS referral pathways, including clear communication to primary care providers and parents about the time-critical nature of NHS follow-up appointments with respect to cCMV.

A further limitation of hearing screening-driven investigations is that infants with asymptomatic cCMV and normal hearing at birth remain undetected. Consequently, these children do not receive the same clinical follow-up as symptomatic neonates, leading to a significant risk of missing or delaying the diagnosis of late-onset hearing loss. Furthermore, the underlying aetiology of cCMV can no longer be verified once the narrow diagnostic window for neonatal testing has passed. Consequently, many hearing impairments developing later in childhood remain classified as hearing loss of unknown aetiology.

Implementing this protocol in routine clinical care would present organisational challenges: timely parental counselling, rapid sample collection coordinated with feeding schedules, and swift referral to confirmatory diagnostics. All require coordinated, multidisciplinary efforts and compliant parents. These demands are challenging, particularly in busy neonatal and paediatric audiology settings. Five improperly obtained buccal swabs for CMV DNA analysis in our study underline the struggles of following study protocol criteria in real-world clinical practice. However, since cCMV is a causally treatable aetiology of congenital hearing loss and timely identification opens a small but crucial window of opportunity for antiviral intervention, these challenges are well worth addressing.

In conclusion, the hearing screening-driven cCMV protocol described herein is well-suited to the systematic and timely identification of newborns with cCMV-associated hearing impairment who may benefit from antiviral therapy, provided they receive their repeat newborn hearing screening within the first 21 days of life. In the absence of universal cCMV screening, this targeted approach represents a pragmatic, evidence-based, and WHO criteria-compliant strategy to extend clinical benefit to affected infants and their families.

## Figures and Tables

**Table 1:** Results of hearing tests and laboratory confirmation diagnostics in per-protocol analysis.

Laboratory Parameters	Patient 1	Patient 2
*CMV DNA oral mucosal swab* [Ct value]	24.97	17.48
*Urine CMV DNA* [IE/mL]	60,871,272	34,387,553
*Plasma CMV DNA* [IE/mL]	<200	2270
*CMV DNA in DBS* [copies/mL]	2430	Negative
*CMV DNA in DBS* [qualitative]	Positive	Positive
*CMV isolated from urine* [nuclei]	500	1000
*Serum CMV IgG* [AE/mL]	1200	1700
*Serum CMV IgM* [AE/mL]	Negative	Negative
*Hearing tests*		
*TEOAEs: right/left*	Abnormal/Normal	Abnormal/Abnormal
*AABR: right/left*	Normal/Normal	Normal/Abnormal

## Data Availability

The data presented in this study are available on request from the corresponding author as they are based on the doctoral thesis of Lena Mistry (née Schütz), which is not available online.
